# Novel strategies to reduce central-line–associated blood stream infection (CLABSI) events in the neonatal intensive care unit

**DOI:** 10.1017/ash.2023.291

**Published:** 2023-09-29

**Authors:** Ingrid Camelo, Srilatha Neshangi, Amy Thompson

## Abstract

**Background:** We describe the components of an improved and easy-to-implement strategy to reduce CLABSI events in the NICU implemented during July–September 2021 in a tertiary-care healthcare center. These strategies were added to an existing institutional protocol created following CDC guidelines. **Methods:** During the previous timeframe of the implementation of new strategies, CDC insertion-related prevention measures [ie, hand hygiene, use of personal protective equipment (PPE), catheter size selection, standard chlorhexidine gluconate (CHG) antisepsis, maintenance related Curos disinfecting caps, and scrubbing the hub] were part of an existing protocol at our institution. We introduced the following key elements along with the previous ones: decrease length of umbilical vein catheter (UVC) utilization from 14 days to 5–7 days, change of dressing materials from BIOPATCH to 3M Tegaderm CHG chlorhexidine gluconate IV securement transparent dressing, enhanced compliance of an existing artificial nail policy, and restricted blood draw from central lines. **Results:** After optimization of the previous protocol through these additional strategies, we achieved a significant reduction in the NICU CLABSI rates from 12 CLABSI events between July 2020 and June 2021 to only 3 CLABSI events between July 2021 and June 2022. **Conclusions:** Revision of CLABSI bundle prevention protocols should be performed frequently to allow improvement opportunities to be added to diminish infection rates. The addition of simple and easy-to-implement key elements interventions to the existing CLABSI bundle had an important impact on the CLABSI rate at our institution.

**Disclosures:** None

